# Guts of healthy humans, livestock, and pets harbor critical-priority and high-risk *Escherichia coli* clones

**DOI:** 10.4178/epih.e2025013

**Published:** 2025-03-22

**Authors:** Idris Nasir Abdullahi, Islem Trabelsi

**Affiliations:** 1 Department of Medical Laboratory Science, Faculty of Allied Health Sciences, College of Medical Sciences, Ahmadu Bello University, Zaria, Nigeria; 2 Bioresources, Environment and Biotechnology Laboratory (LR22ES04), Higher Institute of Applied Biological Sciences of Tunis, University of Tunis El Manar, Tunis, Tunisia

**Keywords:** *Escherichia coli*, One Health, Carbapenem, Colistin, Gastrointestinal microbiome, Cephalosporins

## Abstract

**OBJECTIVES:**

In May 2024, the World Health Organization classified carbapenem (CARB)- and third-generation cephalosporin (3GC) resistance (^R^) in *Escherichia coli* as a critical priority, whereas colistin (COL) is a “last resort” antibiotic for their treatment. This meta-analysis evaluated the pooled prevalence, high-risk lineages, genetic relatedness, and mechanisms of CARB^R^, COL^R^, and 3GC^R^ in *E. coli* from healthy humans and animals.

**METHODS:**

We conducted a systematic review and meta-analyses following the Preferred Reporting Items for Systematic Reviews and Meta-analyses (PRISMA) criteria on all eligible studies that reported the analysis of *E. coli*, and antimicrobial susceptibility to CARB, COL and 3GC in *E. coli* from gut samples of clinically healthy humans, livestock, and pets from June 2014 to June 2024. Random-effect models and conserved signature indels phylogeny 1.4 were used to determine pooled prevalence rates (PPs) and the relatedness of publicly available *E. coli* genomes, respectively.

**RESULTS:**

Of the 5,034 identified articles, 64 studies were deemed eligible. The overall PPs of 3GC^R^, CARB^R^, and COL^R^ *E. coli* were 22.5% (95% confidence interval [CI], 17.5 to 28.3), 2.2% (95% CI, 1.0 to 4.7), and 15.5% (95% CI, 10.8 to 21.8), respectively. The PPs of 3GC^R^-, COL^R^- and CARB^R^ *E. coli* significantly varied by hosts, continent, and year of studies (p<0.05). Diverse *E. coli* lineages were found, including 13 high-risk *E. coli* sequence types (STs), within which ST10 predominated. Phylogenomic analyses produced 4 clusters of related CARB^R^- and COL^R^ *E. coli* strains (<25 single nucleotide polymorphism): ST940-*bla*_OXA-181_ from humans in Lebanon, ST617-*mcr-1* from pigs in China, ST46-*mcr-1* from poultry in Tanzania, and ST1720-*mcr-1* from goats in France.

**CONCLUSIONS:**

COL^R^ and 3GC^R^ are more frequent than CARB^R^ in gut *E. coli*. These 10-year epidemiological data highlight the persistence and transmission of critical priority and high-risk *E. coli* strains in healthy humans and animals, raising significant One Health concerns.

## GRAPHICAL ABSTRACT

[Fig f5-epih-47-e2025013]

## Key Message

• *Escherichia coli* that are resistant to third-generation cephalosporins, carbapenems, and colistin can colonize the guts and potentially cause extra-intestinal infections and increased transmission in healthcare settings, especially if they are high-risk clones.

• To develop effective infection prevention and control measures against these *E. coli* strains, it is essential to determine the global prevalence and extent of their dissemination among different niches and hosts, including healthy humans and animals.

• This meta-analysis of 10-year epidemiological and genomic data found profound persistence and transmission of critical priority and high-risk *E. coli* clones in healthy humans and animals around different locations.

## INTRODUCTION

Ecologically, *Escherichia coli* is a major gut commensal in humans and animals [1]. However, certain strains may express virulence factors and become pathogenic within the gut (e.g., traveler’s diarrhea) or disseminate to other body sites, causing infections such as cholecystitis, pneumonia, neonatal meningitis, sepsis, and urinary tract infections [2,3]. Specifically, extraintestinal pathogenic *E. coli* consists of 4 pathotypes: neonatal meningitis *E. coli*, uropathogenic *E. coli*, avian pathogenic *E. coli*, and septicemic *E. coli* [4]. These classifications are based on the original host and the clinical symptoms produced [4]. Moreover, pathogenic *E. coli* strains in humans and animals can be acquired from contaminated environments, underscoring their relevance within One Health ecosystems [5].

A holistic approach that considers the interconnectedness of human, animal, and environmental health may provide fresh insights into the spread of *E. coli* and demonstrate its capacity to establish in a wide variety of vertebrate hosts, including humans and food-producing animals [6]. Robust evidence shows that pathogenic *E. coli* strains can be transmitted from food-producing animals to humans via food [7]. However, the specific impact of foodborne zoonotic *E. coli* on the overall burden of extraintestinal infections in humans, and the distinct traits that differentiate these strains, remain poorly defined. Recently, reverse zoonosis (transmission from humans to animals) has emerged as a significant issue in infectious disease research due to changes in social environments, such as the increasing number of companion animals and closer interactions with their owners. Livestock farmers or workers may also serve as sources of antibiotic-resistant *E. coli* strains to animals at the farm level [6].

It is important to note that the burden of *E. coli* increases exponentially when it carries critical antimicrobial resistance (AMR) mechanisms against last-resort antibiotics used in clinical settings [8]. Such resistance is a major public health concern when mediated by transferable genes or chromosomal point mutations conferring resistance to third-generation cephalosporins (3GCs), colistin (COL), or carbapenems (CARB) [9]. Specifically, COL sulfate is considered one of the final treatment options for severe infections caused by CARB-resistant (CARB^R^) *E. coli* [10]. Resistance mechanisms may be mediated by transferable genes against COL (*mcr-1* to 11 and *mgrB*) and CARB (*bla*_OXA-48_, *bla*_OXA-244_, *bla*_KPC_, *bla*_SME_, *bla*_NDM_, *bla*_SPM_, *bla*_VIM_, *bla*_GES_, and *bla*_IMI_) [11,12]. Moreover, chromosomal point mutations in *oprD* and *pmrB*/*phoQ* genes have been linked to CARB and COL resistance, respectively [11,12]. Furthermore, some variants of extended-spectrum beta-lactamase (ESBL) genes, *bla*_CTX-M_, *bla*_TEM_, and *bla*_SHV_ mediate 3GC-resistant (3GC^R^) [13].

The World Health Organization (WHO) classifies 3GCs and CARBs as “critically important antimicrobials for human medicine.” However, there has been a significant increase in resistance to these antibiotics [14]. The rise and proliferation of 3GC, CARB, and COL resistance in *E. coli* primarily result from misuse, irrational use or overprescription in human medicine and illegal use in veterinary medicine and animal husbandry [15]. Host colonization and infections in individuals with CARB^R^ or COL-resistant (COL^R^) *E. coli* who have not received these last-resort antibiotics or had contact with settings and hosts that are colonized with critically resistant *E. coli* indicate the evolution and persistence of the resistance genes driven by mobile genetic elements (MGEs) [16,17]. The selection pressure for CARB and polymyxin resistance in gut *E. coli* may vary by host and geographical region [18,19].

Extensive efforts have been made to identify and characterize critically resistant *E. coli* in clinically ill humans and animals; however, data on healthy humans and animals remain scarce. Preserving the efficacy of these 3 antibiotic categories (3GC, CARB, and COL) is vital because they are critical to antimicrobial chemotherapy for *E. coli* infections. Therefore, it is essential to determine whether the gastrointestinal tracts of healthy humans and animals serve as significant ecological niches and transmission pathways for resistance genes and high-risk *E. coli* strains. Global analyses of these priority *E. coli* strains in healthy hosts can inform surveillance and infection prevention and control measures. To assess the public health impact of these emergent *E. coli* strains, we conducted a systematic review and meta-analysis of studies that examined *E. coli* and its susceptibility to 3GC, CARB, and COL in healthy humans, livestock, and companion animals over the past decade (2014-2024). In addition, epidemiological and genomic analyses were performed to elucidate the transmission pathways of critically resistant and high-risk *E. coli* lineages.

## MATERIALS AND METHODS

This systematic review and meta-analysis included original research and brief communications that reported the detection of *E. coli*, its resistance, and resistance mechanisms against 3GC, CARB, and COL in healthy humans, livestock, and pets. The entire process—including literature search strategy, selection of articles, data extraction, and results presentation—adhered to the guidelines outlined in the Preferred Reporting Items for Systematic Reviews and Meta-Analyses (PRISMA; http://prisma-statement.org/PRISMAstatement/check_list.aspx), accessed on October 1, 2024. Articles investigating *E. coli* and its CARB^R^, 3GC^R^, and COL^R^ strains were scrutinized from Scopus, Web of Science, Google Scholar, and PubMed databases. Only original research articles and brief communications published between June 1, 2014 and June 1, 2024 were included. Search terms used included: “*Escherichia coli* fecal carriage in healthy livestock,” “*Escherichia coli* fecal carriage in healthy goats,” “*Escherichia coli* fecal carriage in healthy cattle,” “*Escherichia coli* fecal carriage in healthy sheep,” “*Escherichia coli* fecal carriage in healthy ewe,” “*Escherichia coli* fecal carriage in healthy poultry,” “*Escherichia coli* fecal carriage in healthy chicken,” “*Escherichia coli* fecal carriage in healthy horses,” “*Escherichia coli* fecal carriage in healthy buffaloes,” “*Escherichia coli* fecal carriage in healthy pigs,” “*Escherichia coli* fecal carriage in healthy pets,” “*Escherichia coli* fecal carriage in healthy dogs,” “*Escherichia coli* fecal carriage in healthy cats,” “*Escherichia coli* fecal carriage in healthy companion animals,” “*Escherichia coli* fecal carriage in healthy humans,” “carbapenem-resistant *Escherichia coli* in healthy livestock,” “carbapenem-resistant *Escherichia coli* in healthy pets or companion animals,” “carbapenem-resistant *Escherichia coli* in pets,” “carbapenem-resistant *Escherichia coli* in healthy humans,” “colistin-resistant *Escherichia coli* in livestock,” “colistin-resistant *Escherichia coli* in healthy pets or companion animals,” “colistin-resistant *Escherichia coli* in pets,” “colistin-resistant *Escherichia coli* in healthy humans,” “cephalosporin-resistant *Escherichia coli* in healthy livestock,” “cephalosporin-resistant *Escherichia coli* in healthy pets or companion animals,” “cephalosporin-resistant *Escherichia coli* in healthy pets,” “cephalosporin-resistant *Escherichia coli* in healthy humans,” “ESBL-producing *Escherichia coli* in healthy livestock,” “ESBL-producing *Escherichia coli* in healthy pets or companion animals,” “ESBL-producing *Escherichia coli* in healthy pets,” and “ESBL-producing *Escherichia coli* in healthy humans.”

Out of the initial 5,034 results (4,932 from databases and 102 from registers), 4,880 articles were eliminated as they did not pertain to *E. coli*. Additionally, 90 articles were eliminated due to inadequate methodology, or being review papers (Supplementary Material 1). Following this screening process, 64 studies specifically addressing *E. coli* in healthy humans, pets and livestock were included (Supplementary Materials 1 and 2). Articles solely concentrating on samples from sick or hospitalized humans or animals were excluded. Furthermore, aquatic and wild animals were eliminated because it was difficult to ascertain whether the animals were healthy.

Data from these studies were used to compute the pooled prevalence of 3GC^R^ (cefotaxime, ceftriaxone, cefepime, ceftazidime, cefixime), CARB^R^ and COL^R^ in the pool of non-duplicated *E. coli* strains reported in the individual studies. Only 12 articles met the criteria for further genomic analyses (Supplementary Material 1).

Samples were collected from feces, anal, rectal, and intestinal swabs from healthy humans and animals. Human gut samples were deemed to be from healthy hosts if the methodology explicitly stated “healthy.” Similarly, animal samples were considered eligible if the study indicated the animals were healthy or if the health status description excluded clinical illness. In these studies, the disc diffusion method was most commonly used to determine inhibition zones, although minimal inhibitory concentration testing via broth dilution and E-tests were used for categorizing COL^R^. Specific polymerase chain reaction assays were employed to characterize resistance mechanisms for COL and CARB, and some studies used whole-genome sequencing for further characterization. Genomic data from these strains were obtained from NCBI (https://www.ncbi.nlm.nih.gov/) and were used to determine genetic parameters such as the resistome, sequence types (STs), and plasmid content (Supplementary Material 2).

The conserved signature indels phylogeny database from the Center for Genomic Epidemiology (CGE; https://cge.food.dtu.dk/services/CSIPhylogeny/, accessed October 6, 2024) was used to assess the relatedness of genomes from eligible *E. coli* strains. Single nucleotide polymorphism (SNP) differences among 50 publicly available *E. coli* genomes (Supplementary Material 3) were determined by mapping to the *E. coli* strain K-12 substrain MG1655 reference strain (GenBank accession No. GCA_000005845.2) using default parameters, except that the minimum SNP distance was disabled. Graphical data were incorporated into the phylogenies using iTOL version 6.6. STs were determined using MLST version 2.16 (https://cge.food.dtu.dk/services/MLST/). MGEs and AMR genes were identified using PlasmidFinder and ResFinder from the CGE, while CARD (https://card.mcmaster.ca/analyze/rgi) was used to search for additional AMR genes.

MedCalc version 23.0.2 (MedCalc Software Ltd., Ostend, Belgium) and Comprehensive Meta‐Analysis version 4 (Englewood, NJ, USA) were used for all statistical analyses. All statistical tests were 2-tailed, with p-value <0.05 considered statistically significant. The percentage of 3GC^R^, COL^R^, and CARB^R^ strains was calculated using the formula:

Prevalence of critically resistant *E. coli*=(No. of non-duplicated 3GC^R^, COL^R^, and CARB^R^ strains)/(No. of total non-duplicated *E. coli* strains)

### Ethics statement

No ethical approval is necessary as this is a systematic review article.

## RESULTS

**Figure 1.**
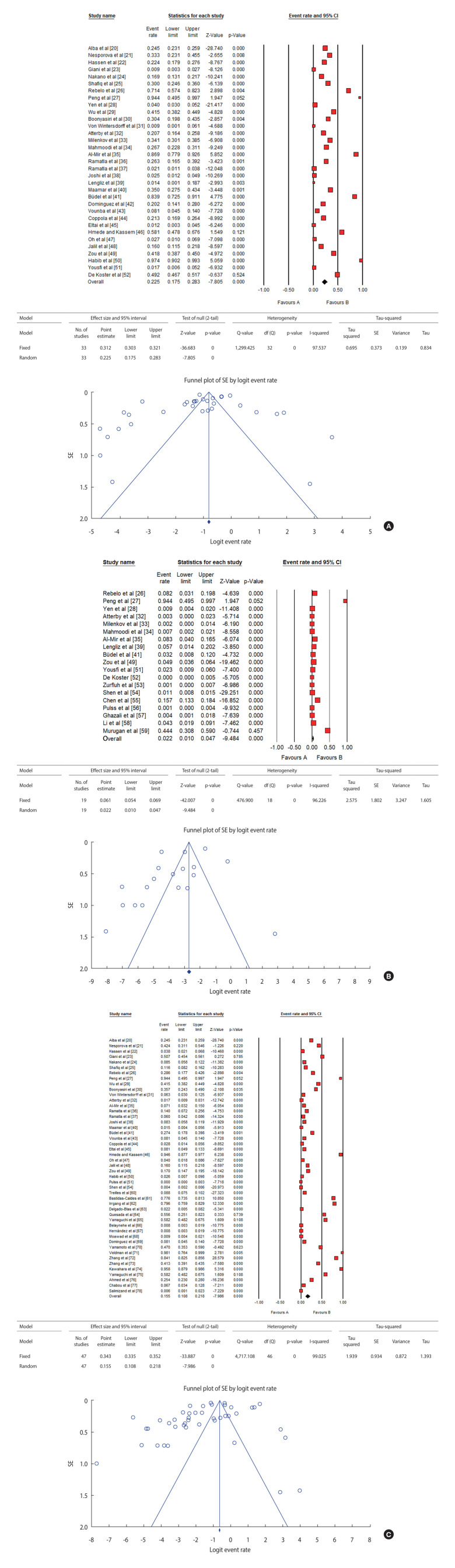
Forest and funnel plot and pooled prevalence of (A) third-generation cephalosporin-resistant, (B) carbapenem-resistant, and (C) colistin-resistant *Escherichia coli* in healthy humans, livestock, and pets. SE, standard error; CI, confidence interval; df, degrees of freedom.

The prevalence of 3GC^R^ *E. coli* from eligible studies ranged from 0.4% (95% confidence interval [CI], 0.2 to 1.0) to 94.4% (95% CI, 49.5 to 99.7) (Figure 1A), with significant heterogeneity among studies (*I*^2^=97.6, p<0.001). The overall pooled prevalence of 3GC^R^ *E. coli* in healthy humans, pets, and livestock was 22.5% (95% CI, 17.5 to 28.3) (Figure 1A) [20-52].

Subgroup analyses showed pooled prevalence rates (PPs) of 3GC^R^ *E. coli* of 12.1% (95% CI, 4.7 to 27.7), 17.8% (95% CI, 13.3 to 23.3), and 41.2% (95% CI, 0.0 to 99.9) in humans, livestock, and pets, respectively. Bivariate analysis showed that livestock had the highest pooled odds of 3GC^R^ *E. coli* (odds ratio [OR], 3.13; 95% CI, 1.97 to 4.99; p<0.001) (Table 1).

**Figure 2.**
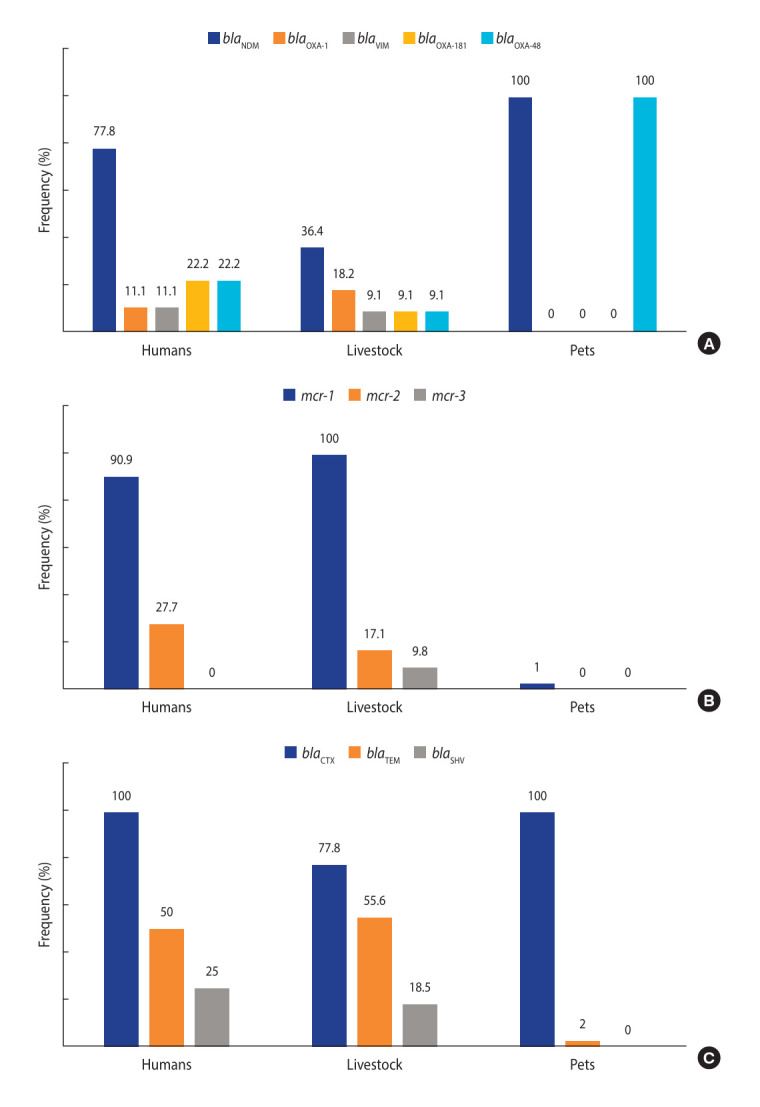
Frequency of studies that reported the mechanisms of (A) carbapenem, (B) colistin, and (C) third-generation cephalosporin resistance in *Escherichia coli* from healthy humans and animals. The number of studies used for computing the frequencies is presented in Table 1.

Data analysis from the present study supports the temporal rise increase of 3GC^R^ *E. coli*. The pooled prevalence of 3GC^R^ *E. coli* from 2020 to 2024 was significantly higher than that from 2014 to 2019 (27.7 vs. 23.2%; OR, 1.27; 95% CI, 1.17 to 1.37, p<0.001) (Table 1). The pooled prevalence of 3GC^R^ *E. coli* in Europe was significantly higher (33.2%; 95% CI, 15.0 to 58.4) than in America (17.9%; 95% CI, 11.7 to 26.4), Asia (16.3%; 95% CI, 10.3 to 24.7) and Africa (15.1%; 95% CI, 8.1 to 26.4) (Table 1). In the 19, 8, and 1 studies on *E. coli* from healthy humans, livestock and pets, respectively, *bla*_CTX-M_ was the most common mechanism of 3GC^R^ (Figure 2).

The overall pooled prevalence of CARB^R^ *E. coli* in healthy humans and animals was 2.2% (95% CI, 1.0 to 4.7) with a significant heterogeneity (*I*^2^=95.7, p<0.001) (Figure 1B) [26-28,32-35,39,41,49,51-59]. Subgroup analyses showed PPs of 1.2% (95% CI, 0.3 to 4.1), 1.5% (95% CI, 0.5 to 4.7), and 2.3% (95% CI, 0.9 to 6.0) in humans, livestock, and pets, respectively (p>0.05) (Table 1). A meta-analysis based on continent showed that Asia (OR, 21.55; 95% CI, 10.16 to 45.70; p<0.001) and Africa (OR, 8.49; 95% CI, 3.16 to 22.89; p<0.001) had significantly higher PPs of CARB^R^ *E. coli* in healthy humans and animals than were observed in Europe (Table 1).

The *bla*_NDM_ gene was the predominant mechanism of resistance in most studies that reported the detection of CARB^R^ *E. coli* in healthy humans and animals (Figure 2). Furthermore, the pooled prevalence was significantly higher from 2014 to 2019 than from 2020 to 2024 (1.4 vs. 1.9%, p<0.001) (Table 1).

The overall pooled prevalence of COL^R^ *E. coli* was estimated to be 15.5% (95% CI, 10.8 to 21.8) with significant heterogeneity (*I*^2^=99.3, p<0.001) (Table 1 and Figure 1C) [20-27,29-32,35-38,40,41,43-51,54,60-78]. Subgroup analyses showed PPs of 13.4% (95% CI, 4.3 to 34.9), 13.0% (95% CI, 7.6 to 21.4), and 6.2% (95% CI, 1.3 to 25.0) in humans, livestock, and pets, respectively (p>0.05) (Table 1). The *mcr-1* gene was found to be the predominant gene responsible for COL resistance among studies that reported the detection of COL^R^ *E. coli* (Figure 2).

The pooled prevalence of COL^R^ *E. coli* was highest in the American continent (24.8%; 95% CI, 11.2 to 46.4), followed by Asia (15.8%; 95% CI, 8.3 to 28.2), but significantly lower in Europe (11.4%; 95% CI, 2.1 to 43.1) and Africa (5.1%; 95% CI, 2.4 to 10.7) (Table 1). As with CARB^R^ *E. coli*, the pooled prevalence of COL^R^ *E. coli* was significantly higher from 2014 to 2019 than from 2020 to 2024 (14.4 vs. 12.0%; OR, 2.42; p<0.001).

**Figure 3.**
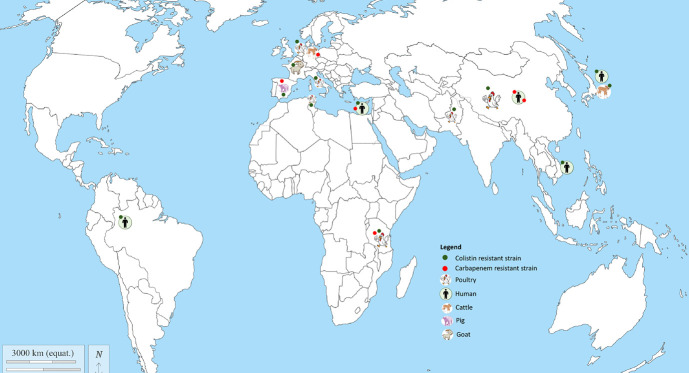
Distribution pattern of colistin- and carbapenem-resistant *Escherichia coli* of the global pandemic sequence type 10 clone.

**Figure 4.**
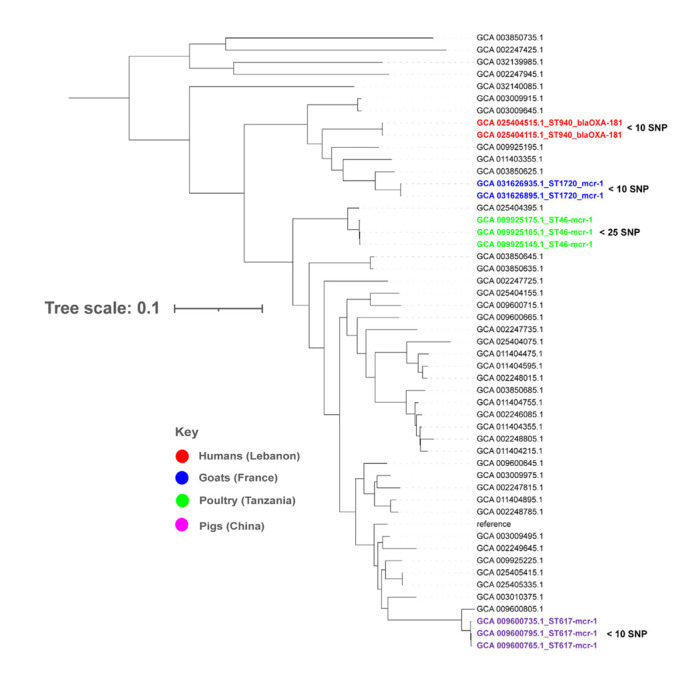
Phylogenomic analysis of 50 publicly available COL-resistant and CARB-resistant *Escherichia coli* mapped against the reference strain, *E. coli* strain. K-12 substrain MG1655 (GenBank accession No. GCA_000005845.2). COL, colistin; CARB, carbapenem; SNP, single nucleotide polymorphism; ST, sequence type.

Diverse lineages (over 100 STs) were reported, including 13 international high-risk *E. coli* clones (ST10, ST38, ST58, ST67, ST69, ST88, ST101, ST131, ST167, ST410, ST457, ST405, ST617, ST648), with ST10 predominating in 17 studies (Table 2) [20-29,53-55,60,71]. COL^R^ and CARB^R^ *E. coli* ST10 were reported on all continents except Oceania (Figure 3). Phylogenomic analyses produced 4 clusters of plasmid-mediated CARB^R^ and COL^R^ *E. coli* strains. High relatedness (<25 SNPs) was observed between *E. coli* strains of ST940-*bla*_OXA-181_ in humans in Lebanon, high-risk ST617-*mcr-1* clone in pigs in China, ST46-*mcr-1* in poultry in Tanzania, and high-risk ST1720-*mcr-1* in goats in France (Figure 4, Supplementary Material 3).

## DISCUSSION

Although *E. coli* are common commensals in the guts of humans and animals, certain extraintestinal strains can cause bloodstream and urinary tract infections [18]. These extraintestinal strains become particularly difficult to treat when they are resistant to 3GC, CARB, and, ultimately, COL. Studies have indicated that pathogenic *E. coli* strains can be transmitted from food-producing animals to humans through contaminated animal-derived food (foodborne zoonotic *E. coli*) [7]. Therefore, it is crucial to determine the trends, global prevalence, and molecular epidemiology of critically resistant *E. coli* in healthy humans, livestock, and pets over the past decade.

Several systematic reviews and meta-analyses on *E. coli* in sick humans and animals have been published. To our knowledge, this is the first comprehensive and simultaneous meta-analysis of critical-priority *E. coli* strains in healthy humans, pets, and livestock. When compared with data from diverse human and animal populations globally, the pooled prevalence of 3GC^R^ *E. coli* in this study is slightly comparable to the 17% (95% CI, 11 to 23) in humans and 22% (95% CI, 9 to 34) in animals in Bangladesh [19]; and the 17.6% (95% CI, 15.3 to 19.8) previously estimated in healthy individuals [79]. However, it is lower than the 21.1% (95% CI, 19.1 to 23.2) reported in inpatients in healthcare settings in a global meta-analysis [80]. These variations may result from differences in the persistence of 3GC^R^, the use of 3GC in patients, and prior selection pressure. Moreover, our study shows a slight decline in global 3GC^R^ *E. coli* prevalence in healthy humans compared to that reported by Bezabih et al. [79]. The discrepancy may be due to the longer study period (January 1, 2000 to April 22, 2021) and larger sample size in the study of Bezabih et al. [79], as opposed to our focus on the last decade (2014-2024). The rise in 3GC^R^ *E. coli* may be attributable to the misuse of antibiotics, including cephalosporins, during the 2021-2023 coronavirus disease 2019 (COVID-19) pandemic, driven by panic over unknown diseases [81]. This misuse might have facilitated the spread and persistence of 3GC^R^ through plasmids in tissues of healthy or asymptomatic individuals [82].

Consistent with previous meta-analyses, *bla*_CTX-M_ was the major mechanism of resistance in 3GC^R^ *E. coli* (Figure 2). For instance, the study of Son et al. [19] reported the predominance of the *bla*_CTX-M_ (70%) gene in ESBL-producing *E. coli*. Moreover, a meta-analysis revealed that 36.3% of all the pooled *E. coli* strains harbored the *bla*_TEM_1 gene in South Africa [83]. Furthermore, *bla*_CTX-M_ was reported to be the most frequent 3GC^R^ gene in a global meta-analysis of *E. coli* strains from swine [84]. It is important to mention that there was no single dominant *bla*_CTX-M_ gene subtype globally, as the subtype varied per country and region. This is not unexpected as CTX-M (cefotaximase-Munich) and TEM (Temoniera) are the major groups of hydrolytic enzymes that are encoded by various ESBL gene variants [85]. In this systematic review, multiple forms of CTX-M-type enzymes coexisted in several studies (Supplementary Material 2), indicating a potentially serious problem in treating ESBL-producing *E. coli* once they disseminate from the gut to other tissues.

The high pooled prevalence of 3GC^R^ *E. coli* in Europe may be explained by the fact that most European studies (75%) focused on healthy livestock (Supplementary Material 2), a subgroup that showed the highest pooled prevalence. In Asia, a pooled prevalence of 48.6% (95% CI, 35.1 to 62.1) of 3GC^R^ *E. coli* was reported in both healthy and sick individuals [86]; this is not surprising given that our study focused solely on healthy humans.

The WHO strongly recommends the urgent development of new antimicrobial agents and control measures against 3GC^R^ and CARB^R^ *E. coli* [8]. Nonetheless, it is essential to determine the global prevalence and extent of dissemination of these strains among different hosts, including healthy humans and animals.

The pooled prevalence of CARB^R^ *E. coli* estimated in this meta-analysis is significantly lower than the 9% reported in Pakistan [87], 9.4% in Nigeria [88], and 29.2% in neonatal sepsis in Africa [89]. These variations likely reflect regional differences in CARB misuse and the endemicity of CARB^R^ *Enterobacterales* [90].

A global meta-analysis reported a 5% pooled prevalence of CARB^R^ *E. coli* in swine [84], which is higher than the 1.5% pooled prevalence obtained from all livestock data in this study. This discrepancy may be explained by the fact that Hayer et al. [84] focused on pigs—the major reservoir of critical resistance genes in *E. coli*—and included samples from both sick and healthy swine.

The unexpectedly higher CARB^R^ *E. coli* prevalence in pets and livestock is notable, especially since CARB antibiotics are not licensed for animal use. CARB antibiotics are rarely prescribed for severely ill humans in some countries due to the complexity of multidrug-resistant Gram-negative bacterial infections [91]. This situation suggests that animals may acquire CARB^R^ *E. coli* strains from their owners or farmers, and vice versa [92,93]. Additionally, the higher rate of CARB^R^ *E. coli* in healthy animals might indicate greater bacterial persistence in their guts compared to humans [94].

First, the differences among continents likely reflect varying levels of CARB misuse. In European countries, stringent regulations restrict the use of these drugs to human medicine. Second, the lower prevalence of CARB^R^ in *E. coli* from 2020-2024 may be attributed to increased awareness and antimicrobial stewardship programs regarding the public health dangers of CARB use, especially in Europe and North America [95].

The pooled prevalence of COL^R^ *E. coli* in this study differs from 28% reported in poultry in Southern Asian countries [96], 5.7% from food and livestock samples [97], and 7.6% among nosocomial strains in India [98]. These variations underscore differences in antibiotic selection pressure, antibiotic use policies, and host susceptibility across countries and sample types. Nevertheless, the high pooled prevalence of COL^R^ *E. coli* across all study groups underscores the diminishing effectiveness of COL as a last-resort antimicrobial agent. The ubiquitous presence of the *mcr-1* gene in all COL^R^ *E. coli* strains from the eligible studies further emphasizes the need for robust monitoring and surveillance programs across all hosts, including healthy ones.

This rapidly emerging global pandemic clone was reported in healthy humans, livestock, and pets (Figure 3).

Several studies have documented the detection of 13 different pandemic *E. coli* clones, highlighting the importance of understanding the diversity and epidemiology of gut *E. coli* with extra-intestinal genetic backgrounds in healthy humans and animals. These high-risk clones are associated with increased virulence, AMR, and a propensity to cause outbreaks in both healthcare and community settings. Although these high-risk *E. coli* strains have been identified in healthy hosts, there is a high likelihood that they could cause infections if translocated from the gut to other tissues or organs.

The diversity and co-carriage of resistance genes—encompassing CARB and 3GC^R^, COL, and 3GC^R^, or even resistance to all 3 antibiotic categories within a single *E. coli* strain (Supplementary Material 2)—demonstrate the complexity of AMR and could undermine current control measures.

This systematic review and meta-analysis synthesized data from previously published studies to generate pooled prevalence estimates. However, the study has limitations. First, there was substantial heterogeneity among the included studies, and subgroup and meta-regression analyses did not fully account for this variability. For example, PPs of 3GC^R^ *E. coli* in livestock may vary among different livestock species, so some prevalence data cannot be generalized. Second, some studies included in the meta-analysis involved a small number of bacterial strains. Caution is also warranted in interpreting some results due to disproportionate distributions of studies by country and host; for instance, only 2 studies involved healthy pets, and no eligible study was found from the Oceania region.

## CONCLUSION

COL^R^ and 3GC^R^ are significantly more frequent than CARB^R^ in gut *E. coli*. This decade-long epidemiological data underscores the persistence and transmission of critical-priority, high-risk *E. coli* strains among healthy humans and animals. Consequently, individuals may acquire these strains through occupational exposure to livestock or direct contact with companion animals. Although healthy humans and animals may remain asymptomatic despite harboring these critically resistant, high-risk clones, their potential to transmit these superbugs to immunocompromised individuals is concerning.

Furthermore, this review enhances our understanding of the molecular epidemiology of critical-priority *E. coli* in healthy hosts, highlighting the importance of molecular surveillance for 3GC^R^, CARB^R^, and COL^R^ *E. coli*. As these high-risk strains become more established in healthy populations, future public health strategies should emphasize a One Health approach supported by genomic sequencing technologies.

## Figures and Tables

**Figure f5-epih-47-e2025013:**
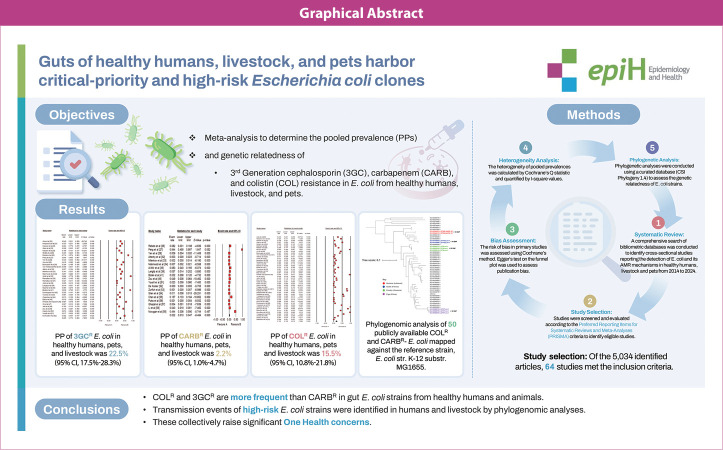


**Table 1.** Pooled prevalence rates of CARB^R^, COL^R^, and 3GC^R^ *E. coli* strains in health status humans and animals by continent and temporal period

Category	Variables	No. of studies pooled	Pooled prevalence % (95% CI)	Heterogeneity *I*^2^ (%)	Cochrane p-value	OR (95% CI)	p-value
CARB^R^ *E. coli*	Hosts						
	All host	19	2.2 (1.0, 4.7)	96.2	<0.001	-	-
	Humans	9	1.2 (0.3, 4.1)	97.7	<0.001	0.95 (0.34, 2.55)	0.898
	Livestock	11	1.5 (0.5, 4.7)	90.7	<0.001	0.43 (0.15, 1.18)	0.101
	Pets	1	2.3 (0.9, 6.0)	100	<0.001	1.00 (reference)	
	Continent						
	Africa	4	2.0 (0.7, 6.1)	62.0	0.050	8.49 (3.16, 22.89)	<0.001
	Asia	11	3.0 (1.2, 7.4)	97.1	<0.001	21.55 (10.16, 45.70)	<0.001
	America	8	0	-	-	-	-
	Europe	4	0.2 (0, 4.6)	92.6	<0.001	1.00 (reference)	
	Temporal trend						
	2020-2024	11	1.9 (0.8, 4.4)	94.6	<0.001	0.24 (0.18, 0.32)	<0.001
	2014-2019	8	1.4 (0.4, 4.8)	89.2	<0.001	1.00 (reference)	
COL^R^ *E. coli*	Hosts						
	All hosts	47	15.5 (10.8, 21.8)	99.0	<0.001	-	-
	Humans	11	13.4 (4.3, 34.9)	96.9	<0.001	2.31 (0.72, 7.42)	0.158
	Livestock	41	13.0 (7.6, 21.4)	99.3	<0.001	3.137 (0.98, 10.03)	0.053
	Pets	2	6.2 (1.3, 25.0)	43.2	0.185	1.00 (reference)	
	Continent						
	Africa	7	5.1 (2.4, 10.7)	90.8	<0.001	0.65 (0.52, 0.83)	<0.001
	Asia	25	15.8 (8.3, 28.2)	99.4	<0.001	2.49 (2.28, 2.71)	<0.001
	America	8	24.8 (11.2, 46.4)	97.8	<0.001	9.6 (8.43, 10.93)	<0.001
	Europe	9	11.4 (2.1, 43.1)	99.4	<0.001	1.00 (reference)	
	Temporal trend						
	2020-2024	17	12.0 (6.7, 20.6)	98.5	<0.001	2.42 (2.22, 2.64)	<0.001
	2014-2019	31	14.4 (7.1, 27.0)	99.4	<0.001	1.00 (reference)	
3GC^R^ *E. coli*	Hosts						
	All hosts	33	22.5 (17.5, 28.3)	97.5	<0.001	-	-
	Humans	8	12.1 (4.7, 27.7)	97.9	<0.001	1.36 (0.84, 2.18)	0.209
	Livestock	27	17.8 (13.3, 23.3)	97.8	<0.001	3.13 (1.97, 4.99)	<0.001
	Pets	2	41.2 (0, 99.9)	95.3	<0.001	1.00 (reference)	
	Continent						
	Africa	9	15.1 (8.1, 26.4)	95.5	<0.001	0.46 (0.41, 0.53)	<0.001
	Asia	16	16.3 (10.3, 24.7)	97.3	<0.001	0.51 (0.47, 0.55)	<0.001
	America	5	17.9 (11.7, 26.4)	86.9	<0.001	0.47 (0.39, 0.56)	<0.001
	Europe	4	33.2 (15.0, 58.4)	99.4	<0.001	1.00 (reference)	
	Temporal trend						
	2020-2024	18	27.7 (26.6, 28.8)	98.2	<0.001	1.27 (1.17, 1.37)	<0.001
	2014-2019	14	23.2 (22.2, 24.2)	94.5	<0.001	1.00 (reference)	

CARB^R^, carbapenem-resistant; COL^R^, colistin-resistant; 3GC^R^, third-generation cephalosporin resistance; OR, odds ratio; CI, confidence interval.

**Table 2.** Distribution pattern of other non-ST10 *Escherichia coli* pandemic clones carrying colistin and carbapenem resistance genes by hosts and countries

Reference	Host	Country	No. of strains/No. of hosts tested	Critical resistance gene
ST131				
Alba et al. [20]	Turkeys	Italy	1/39	*mcr-1.1*
ST38				
Alba et al. [20]	Turkeys	Italy	1/39	*mcr-1.1*
Veldman et al. [71]	Broiler chickens	Netherlands	1/26	*mcr-1*
Nesporova et al. [21]	Broiler chickens	Paraguay	1/28	*mcr-5*
Zurfluh et al. [53]	Humans	Switzerland	1/1	*bla*_OXA-48_
Shen et al. [55]	Humans	China	1/3,859	*bla*_NDM-1_
ST67				
Hassen et al. [22]	Chickens	Tunisia	2/11	*mcr-1*
Giani et al. [23]	Chickens	Bolivia	1/16	*mcr-1*
Nakano et al. [24]	Humans and livestock	Japan	1/28	*mcr-1*
ST410 (CC23)				
Shafiq et al. [25]	Sheep	Pakistan	2/75	*mcr-1*
Nakano et al. [24]	Humans and livestock	Japan	1/28	*mcr-1*
Alba et al. [20]	Turkeys and pigs	Italy	2/39	*mcr-1.1, mcr-4.2*
Veldman et al. [71]	Broiler chickens	Netherlands	2/26	*mcr-1*
Chen et al. [55]	Humans	China	1/758	*bla*_NDM-1_
Rebelo et al. [26]	Pigs	Germany	1/49	*mcr-4*
ST457				
Nesporova et al. [21]	Chickens	Paraguay	12/28	*mcr-5*
Rebelo et al. [26]	Pigs	Germany	1/49	*mcr-4.2*
ST69				
Giani et al. [23]	Humans	Bolivia	1/337	*mcr-1*
Treilles et al. [60]	Goats	France	3/1,561	*mcr-1*
Alba et al. [20]	Livestock	Italy	2/3,521	*mcr-1.13*
Hassen et al. [22]	Chickens	Tunisia	2/286	*mcr-1*
Shafiq et al. [25]	Livestock	Pakistan	1/250	*mcr-1*
Nakano et al. [24]	Calves	Japan	1/202	*mcr-1*
ST617 (CC10)				
Chen et al. [55]	Humans	China	5/758	*bla*_NDM-1_, *bla*_NDM-5_
Peng et al. [27]	Pigs	China	4/8	*bla*_NDM-1_, *mcr-1*
Nakano et al. [24]	Calves	Japan	1/202	*mcr-1*
ST88 (CC23)				
Shafiq et al. [25]	Livestock	Pakistan	1/250	*mcr-1*
Nakano et al. [24]	Calves	Japan	1/202	*mcr-1*
ST167 (CC10)				
Chen et al. [55]	Humans	China	13/758	*bla*_NDM-5_
Shen et al. [54]	Humans	China	5/3,859	*bla*_NDM-5_
Treilles et al. [60]	Goats	France	4/1,561	*mcr-1*
ST405				
Yen et al. [28]	Humans	Vietnam	1/652	*bla*_NDM-1_
ST648				
Rebelo et al. [26]	Calves	France	1/49	*mcr-1*
Veldman et al. [71]	Calves	Netherlands	4/15	*mcr-1*
ST101				
Shen et al. [54]	Humans	China	1/3,859	*bla*_NDM-5_
Alba et al. [20]	Turkeys	Italy	2/39	*mcr-1.1*
Wu et al. [29]	Chickens	China	3/821	*mcr-1*
ST58				
Shen et al. [54]	Humans	China	1/3,859	*bla*_NDM-5_
Shafiq et al. [25]	Livestock	Pakistan	1/250	*mcr-1*

ST, sequence type; CC, clonal complex.

## Supplementary materials

Supplementary Material 1. Literature search, inclusion and exclusion processes.

Supplementary Material 2. Critical resistance mechanisms, mobile genetic elements, and lineage of *E. coli* strains from eligible studies on healthy humans and animals

Supplementary Material 3. SNP difference between 50 publicly available genomes of carbapenem and costin resistant *E. coli* strains from healthy huamns and animals
