# Supplementary material for: Guts of healthy humans, livestock, and pets harbor critical-priority and high-risk Escherichia coli clones
Source: Epidemiol Health. 2025 Mar 22;47:e2025013. doi: 10.4178/epih.e2025013 (PMC12425698; doi:10.4178/epih.e2025013)
Supplement: Supplementary Material 1. — Literature search, inclusion and exclusion processes. [file epih-47-e2025013-Supplementary-1.docx]

**Identification of studies via databases and registers**

Records removed before screening:

Duplicate records removed (n = 3701)

Records marked as ineligible by automation tools (n = 752)

Records removed for other reasons (n =27)

Records identified from:

Databases (n = 4932)

Registers (n = 102)

**Identification**

Records screened

(n = 154)

Records excluded

(n = 67)

Reports sought for retrieval

(n = 87)

Reports not retrieved

(n =3)

**Screening**

Reports assessed for eligibility

(n =84)

Reports excluded:

Case reports (n = 5)

Duplicated data (n =14)

Incomplete or inaccurate data (n =1)

- Studies included in meta-analyses = 64)
- Studies included for phylogenomic analyses = 12

**Included**

**Supplementary Material 1**. Literature search, inclusion and exclusion processes.
