# Supplementary material for: Guts of healthy humans, livestock, and pets harbor critical-priority and high-risk Escherichia coli clones
Source: Epidemiol Health. 2025 Mar 22;47:e2025013. doi: 10.4178/epih.e2025013 (PMC12425698; doi:10.4178/epih.e2025013)
Supplement: Supplementary Material 2. — Critical resistance mechanisms, mobile genetic elements, and lineage of E. coli strains from eligible studies on healthy humans and animals [file epih-47-e2025013-Supplementary-2.docx]

**Supplementary Material 2.** Critical resistance mechanisms, mobile genetic elements, and lineage of E. coli strains from eligible studies on healthy humans and animals

| Authors | Country | Type of host (number) | Critical resistance Phenotype (number of strains) | Molecular Assays used | Mechanisms of resistance | Genetic Lineage | Locations of COL^R^ and CARB^R^ genes |
| --- | --- | --- | --- | --- | --- | --- | --- |
| Healthy Humans | | | | | | | |
| Li *et al* [1] | China | Humans (141) | CARB (6) | WGS | *bla*_NDM_ | ST1011, ST48 | Not in plasmid |
| Boonyasiri *et al* [2] | Thailand | Humans (28) | COL (3), CTX (10) | WGS | *mcr-1.1, mcr-3.5, mcr-3.1* *bla*_CTX-M-55_, *bla*_TEM-1_ | ST7626, ST3045, ST354 | *IncX4, IncFII* |
| Salimizand *et al* [3] | Iran | Humans (339) | COL (2) | PCR | *mcr-1* | NT | NT |
| Yen *et al* [4] | Vietnam | Humans (652) | CARB (4), FEP (3), CTX (4), CAZ (3), CRO (3) | WGS | *bla*_NDM-1_, *bla*_OXA-1_, *bla*_OXA-181_, *bla*_TEM_, *bla*_CTX_ | ST405*, ST1638, ST38*, ST2705 | Not in plasmid |
| Bastidas-Caldes *et al* [5] | Ecuador | Humans (137) | COL (111), CTX (20) | PCR | *mcr-1* | NT | NT |
| Von Wintersdorff *et al* [6] | Netherlands | Humans (112) | COL (7), CTX (1) | PCR | *mcr-1*, *bla*_CTX-M-2_, *bla*_CTX-M-9_ | ST80, ST744, ST1011 | NT |
| Yamaguchi *et al* [7] | Vietnam | Humans (98) | COL (69) | WGS | *mcr-1* | ST206, ST48, ST10*, ST46, ST189, ST590, ST165, ST155, ST201, ST189, ST542, ST206-like | Seven strains carry *mcr-1 gene* in chromosomes the rest in *IncX4, IncHI2* |
| Atterby *et al* [8] | Cambodia | Humans (307) | COL (2), CARB (1) | PCR | *bla*_OXA-48_, *mcr-1, mcr-3,* *bla*_CTX-M-15_, *bla*_CTX-M-14_, *bla*_CTX-M-27_, *bla*_CTX-M-55_, *bla*_SHV-2_, *bla*_SHV-12_, *bla*_SHV-28_, *bla*_CMY-2_, *bla*_CMY-42_, *bla*_DHA-1_ | NT | NT |
| Yamamoto *et al* [9] | Vietnam | Humans (98) | COL (68) | PCR | *mcr-1, mcr-3, mcr-1/3* | NT | NT |
| Milenkov *et al* [10] | Madagascar | Humans (492) | CARB (1), CTX (168), CAZ (105), FEP (124) | WGS | *bla*_NDM−5_, *bla*_CTX-M1_, *bla*_CTX-M-9_, *bla*_CTX-M-2_ | ST1312 | *IncF, IncY* |
| Zurfluh *et al* [11] | Switzerland | Humans (1086) | CARB (1) | PCR | *bla*_OXA-48_, *bla*_CTX-M-24_, *bla*_TEM-1_ | ST38* | NT |
| Giani *et al* [12] | Bolivia | Humans (337) | COL (171), CTX (3), CAZ (3), FEP (3) | WGS | *mcr-1,* *bla*_TEM-1A_, *bla*_TEM-1B_, *bla*_CTX-M-55_ | ST48, ST744, ST10*, ST206, ST2705, ST2936, ST1286, ST7570, ST69*, ST117, ST711, ST7571, ST3056 | *Incl2, IncHI1* |
| Chen *et al* [13] | China | Humans (758) | CARB (119) | WGS | *bla*_NDM-1_, *bla*_NDM-4_, *bla*_NDM-5_, *bla*_NDM-7_, *bla*_NDM-9_, *bla*_NDM-13_ | ST10*, ST46, ST48, ST167*, ST206, ST617*, ST746, ST5048, ST6395, ST8666, ST8667, ST34, ST155, ST156, ST165, ST180, ST181, ST189, ST195, ST218, ST224, ST226, ST297, ST359, ST361, ST410*, ST656, ST711, ST744, ST770, ST1695, ST1788, ST2854, ST3274, ST1011, ST1114, ST3856, ST3896, ST4429, ST4537, ST4542, ST5044, ST6335, ST6395, ST6449, ST6727, ST6793, ST6910, ST7331, ST7366, ST8153, ST8668, ST8670, ST10170 | Not in plasmid |
| Shen *et al* [14] | China | Humans (3859) | COL (14), CARB (43), CTX (57), CAZ (57), FEP (53) | WGS | *mcr-1,* *bla*_NDM-5_, *bla*_NDM-1_ | ST167*, ST10*, ST744, ST7153, ST48, ST7386, ST746, ST6793, ST206, ST524, ST7387, ST6395, ST7402, ST189, ST2705, ST6607, ST354, ST408*, ST38*, ST410*, ST58*, ST155, ST3759, ST224, ST2973, ST1324, ST101*, ST156 | a. *IncX3* carrying *bla*_NDM-5_  b. *IncHI2* carried *mcr-1* |
| Mahmoodi *et al* [15] | Iran | Humans (430) | CARB (3), CTX (48), CAZ (115) | PCR | *bla*_NDM-1_, *bla*_VIM-2_ | NT | NT |
| Al-Mir *et al* [16] | Lebanon | Humans (84) | COL (6), CARB (7), CTX (73) | WGS | *mcr-1.1*, *bla*_OXA-181_, *bla*_OXA-244_, *bla*_NDM-5_, *bla*_CTX-M-15_, *bla*_CTX-M-27_, *bla*_CTX-M-3_, *bla*_CMY-42_, *bla*_CMY-2_, *bla*_DHA-1_, *bla*_SHV-12_, *bla*_VEB-1_ | ST46, ST540, ST940, ST1722, ST6836, ST10*, ST56, ST1303 | a*. IncX3* and *IncFI* carried *bla*_OXA-181_  b*. IncI1* carried *bla*_OXA-244_*,*  c. *IncX3* carried *bla*_NDM-5_ |
| Nakano *et al* [17] | Japan | Humans (62) | COL (3) | PCR | *mcr-1*, *bla*_CTX-M-27_, *bla*_CTX-M-156_ | ST10*, ST746, ST2929 | NT |
| Healthy Livestock | | | | | | | |
| Ramatla *et al* [18] | South Africa | Sheep (37), goats (20) | COL (8), CTX (15) | PCR | *mcr-1, mcr-2, bla*_CTX−M−15_, *bla*_CTX−M−25_, *bla*_CTX−M−9_, *bla*_CTX−M−2_, *bla*_TEM_, *bla*_SHV_. | NT | NT |
| Ramatla *et al* [19] | South Africa | Chicken (480) | COL (29), CTX (10) | PCR | *bla*_CTX-M-9_, *bla*_CTX-M-25_, *bla*_CTX-M-2_, *bla*_SHV_, *bla*_CARB_, *bla*_TEM_, *bla*_CTX-M-15_, *bla*_CTX-M_ | NT | NT |
| Boonyasiri *et al* [20] | Thailand | Pigs (38) | COL (17), CTX (7) | WGS | *mcr-1.1, mcr-3.5, mcr-3.2, mcr-2.3, mcr-3.4, mcr-3.1, mcr-3.24,* *bla*_CTX−M−55_, *bla*_TEM-1_, *bla*_CTX−M−14_ | ST206, ST34, ST165, ST1602, ST1040, ST5218, ST398, ST34, ST4429, ST7625, ST48, ST10* | *IncX4, IncFII* carried all the COL^R^ gene |
| Salimizand *et al* [21] | Iran | Poultry (400) | ND | PCR | NA | NT | NT |
| Treilles *et al* [22] | France | Goats (1701) | COL (149) | WGS | *mcr-1,* *bla*_CTX-M-15_, *bla*_TEM_ | ST69*, ST1011, ST10*, ST167*, ST215, ST744, ST940, ST1720, ST362, ST57, ST5841, ST591, ST301, ST973, ST17, ST162, ST1, ST3014, ST164, ST2, ST448, ST1642, ST4981, ST3902, ST224, ST997 | a. *IncX4* and *IncHI2* carried *mcr-1*in 135 strains  b. 13 strains had the *mcr-1* gene in chromosomes  c. One strain has unknown location of the *mcr-1* gene |
| Yen *et al* [23] | Vietnam | Chickens (237), ducks (150), pigs (143) | CARB (1), FEP (1), CTX (43), CAZ (1), CRO (1) | WGS | *bla*_NDM-1_, *bla*_TEM,_ | ST398 | Not in plasmid |
| Bastidas-Caldes *et al* [24] | Ecuador | Chickens (154), pigs (147) | COL (229) | PCR | *mcr-1* | NT | NT |
| Peng *et al* [25] | China | Pigs (8) | COL (8), CARB (8), CAZ (8), CRO (8), FEP (8) | WGS | *bla*_NDM-1_, *mcr-1,* *bla*_TEM-1B_, *bla*_CTX-M-14_ | ST617*, ST746, ST695, ST7050 | *bla*_NDM-1_ in *IncFII,* while *mcr-1* in *IncX4* |
| Irrgang *et al* [26] | Germany | Chicken, turkey, cattle, pig (505) | COL (402) | PCR | *mcr-1* | NT | NT |
| Delgado-Blas *et al* [27] | Venezuela | Swine (93) | COL (2), CTX (1) | WGS | *mcr-1* | ST452 | Located in *IncFIB, IncI2, ColpVC, Col8282* |
| Rebelo *et al* [28] | Spain  Germany, France and Italy | Pigs and calves (49) | COL (14), CARB (4), CTX (35) | WGS | *bla*_OXA-1_, *mcr-1, mcr-3, mcr-4,* *bla*_CTX_, *bla*_TEM_ | ST410*, ST224, ST950, ST10*, ST457*, ST533, ST744, ST648*, ST5995, ST4096 | *mcr* genes carried by *ColE10, Col156, Col8282, ColRNAI, IncFII, IncFIA, IncFIB* |
| Quesada *et al* [29] | Spain | Turkey (170), swine (439) | COL (5) | PCR | *mcr-1* | NT | NT |
| Joshi *et al* [30] | Nepal | Chicken (324) | COL (27), CTX (8), CAZ (1) | PCR | *mcr-1,* *bla*_CTX-M_ | NT | NT |
| Pulss *et al* [31] | Germany and Italy | Pigs (2253) | COL (1), CARB (2) | WGS | *mcr-1,* *bla*_OXA-48_-like, *bla*_OXA-181_ | ST359, ST641 | *mcr-1* located in *IncX4,* while *bla*_OXA-181_ in *IncX3* |
| Lengliz *et al* [32] | Tunisia | Rabbits (35) | CARB (2) | PCR | *bla*_VIM_, *bla*_IMP_ | NT | NT |
| Ghazali *et al* [33] | Malaysia | Ruminants (151), swine (100), broiler chicken (200) | CARB (2) | PCR | *bla*_NDM_ | NT | NT |
| Belaynehe *et al* [34] | South Korea | Cattle (341), swine (265), chicken (30) | COL (5) | PCR | *mcr-1, mcr-3* | NT | NT |
| Hernández *et al* [35] | Spain | Cattle (636) | COL (5) | WGS | *mcr-1, mcr-3,* *bla*_CTX-M-55_, blaTEM-1A | ST533 | *mcr-1* and *mcr-3* carried by *IncHI2, IncI1-1_Alpha* |
| Kawanishi *et al* [36] | Japan | Cattle (3134), swine (2052), broilers (2017), layers (2103) | COL (39) | PCR | *mcr-1* | NT | NT |
| Alba *et al* [37] | Italy | Chickens, turkeys, pigs, bovines (3521) | COL (42), CTX (862) | WGS | *mcr-1.1, mcr-1.2, mcr-1.13, mcr3.2, mcr-4.2, mcr-4.3* | ST131*, ST155, ST156, ST744, ST101*, ST10*, ST410*, ST69*, ST5995, ST38* | *IncFIB, IncX4, IncFIC(FII), IncX1, Col156, IncR, IncFII, IncFI, IncHI2, IncI, IncN* |
| Wu *et al* [38] | China | Chickens (821) | COL (44), CTX (341) | WGS | *mcr-1,* *bla*_CTX-M-55_, *bla*_CTX-M-65_, *bla*_TEM_ | ST46, ST1286, ST10*, ST29, ST101*, ST354 | *mcr-1* carried by *Incl2* |
| Maamar *et al* [39] | Tunisia | Chickens (137) | COL (2), CTX (48) | PCR | *mcr-1,* *bla*_CMY-2_ | ST2197 | NT |
| Moawad *et al* [40] | Egypt | Chickens (576) | COL (5), CAZ (23) | PCR | *mcr-1* | NT | NT |
| Hassen *et al* [41] | Tunisia | Chickens (286) | COL (11), CTX (64) | PCR | *mcr-1, bla*_TEM-1_, *bla*_CTX-M-55_, *bla*_CTX-M-14_, *bla*_CTX-M-1_, *bla*_CMY-2_ | ST57, ST69*, ST162, ST2220, ST10*, ST5686, ST997, ST6488, ST6789 | NT |
| Atterby *et al* [8] | Cambodia | Ruminants, pigs, poultry (285) | COL (8), CTX (59) | PCR | *mcr-1, mcr-3,* *bla*_CTX-M-15_, *bla*_CTX-M-14_, *bla*_CTX-M-27_, *bla*_CTX-M-55_, *bla*_SHV-2_, *bla*_SHV-12_, *bla*_SHV-28_, *bla*_CMY-2_, *bla*_CMY-42_, *bla*_DHA-1_ | NT | NT |
| Büdel *et al* [42] | Tanzania | Poultry (62) | COL (17), CARB (2), CTX (52) | WGS | *mcr-1,* *bla*_OXA-1_, *bla*_CTX-M-15_, *bla*_CTX-M-9_ | ST10*, ST1585, ST46, ST224, ST155 | *mcr-1* carried by *IncFIB, IncX4,* and *IncFII* |
| Dominguez *et al* [43] | Argentina | Broilers (129) | COL (31), CTX (26), CRO (26), FEP (24), CAZ (5) | PCR | *mcr-1*, *bla*_CTX-M-2_, *bla*_CTX-M-14_, *bla*_CMY-2_ | NT | NT |
| Vounba *et al* [44] | Canada, Senegal and Vietnam | Chickens (136) | COL (11), CRO (13), CTX (11) | PCR | *mcr-1,* *bla*_CTX-M_, *bla*_TEM_, *bla*_CMY-2_ | NT | NT |
| Yamamoto *et al* [45] | Ecuador | Pigs (34), chickens (32) | COL (31) | PCR | *mcr-1* | NT | NT |
| Nesporova *et al* [46] | Paraguay | Chickens (66) | COL (28), CTX (22) | PCR | *mcr-5*, *bla*_CTX-M-8_, *bla*_CMY-2_, *bla*_SHV-12_ | ST457*, ST38, ST57, ST8061, ST224, ST366, ST641, ST752, ST165, ST189, ST580, ST6853, ST93, ST2705, ST38* | NT |
| Coppola *et al* [47] | Uruguay | Chickens (132), calves (100), pigs (50) | COL (8), CRO (60) | PCR | *mcr-1,* *bla*_CTX-M-8,_ *bla*_CMY-2,_ *bla*_CTX-M-2_, *bla*_SHV-12_, *bla*_CTX-M-14_, *bla*_CTX-M-15_, *bla*_SHV2a_, *bla*_CTX-M-55_ | NT | NT |
| Eltai *et al* [48] | Qatar | Poultry (172) | COL (14), CRO (2), FEP (1) | PCR | *mcr-1* | NT | NT |
| Hmede and Kassem [49] | Lebanon | Chickens (93) | COL (88), CTX (54), FEP (21) | WGS | *mcr-1,* *bla*_TEM_, *bla*_CTX-M_ | NT | Not in plasmid |
| Veldman *et al* [50] | Netherlands | Calves (15), broilers (10), turkey (1) | COL (26) | PCR | *mcr-1* | ST1730, ST4512, ST752, ST1564, ST57, ST10*, ST410*, ST648*, ST624, ST1011, ST38*, ST351, ST2309 | NT |
| Lv *et al* [51] | Pakistan | Broilers (100) | COL (8) | PCR | *mcr-1* | ST10*, ST2847, ST155, ST361, ST6395, 2 new STs | NT |
| Zhang *et al* [52] | China | Pigs (811), chickens (1232), cattle (156) | COL (1850) | PCR | *mcr-1, mcr-2* | NT | NT |
| Zhang *et al* [53] | China | Pigs, poultry (chicken, duck, goose, pigeon) (1922) | COL (794) | PCR | *mcr-1, mcr-2, mcr-3* | NT | NT |
| Oh *et al* [54] | South Korea | Chickens (34), pigs (59), cattle (57) | COL (6), CTX (4) | PCR | *mcr-1*, *bla*_TEM-1_, *bla*_CTX-M-1_, *bla*_CTX-M-55_ | ST898, ST2705, ST6706, ST5229 | NT |
| Kawahara *et al* [55] | Vietnam | Pigs (36), chickens (36) | COL (69) | PCR | *mcr-1, mcr-3* | NT | NT |
| Jalil *et al* [56] | Pakistan | Cows (200) | COL (32), CTX (32), CAZ (12), CRO (28), FEP (90) | PCR | *mcr-1,* *bla*_TEM_, *bla*_CTX_ | NT | NT |
| Zou *et al* [57] | China | Chickens (926) | COL (157), CARB (45), CTX (387) | PCR | *mcr-1, bla*_NDM_, *bla*_CTX-M-1G_, *bla*_CTX-M-9G_ | ST117, ST93, ST569, ST1485, ST2944, 1 new ST | NT |
| Ahmed *et al* [58] | Bangladesh | Chickens (1200) | COL (305) | WGS | *mcr-1,* *bla*_TEM_ | ST43, ST4965 | *ColRNAI, IncFIB, IncHI2, IncHI2A, IncN, IncX1, IncI2* |
| Chabou *et al* [59] | Algeria | Chickens (120) | COL (8) | PCR,  qPCR | *mcr-1* | ST48 | NT |
| Senthil Murugan *et al* [60] | India | Cattle (45) | CARB (20) | ND | NT | NT | NT |
| De Koster et al [61] | Belgium and Netherlands | Broilers (779) and pigs (817) | CARB (0), CTX (757) | NT | NT | NT | NT |
| Shafiq *et al* [62] | Pakistan | Buffaloes, cattle, sheep, goats, broilers (250) | COL (29), CTX (75) | PCR | *mcr-1,* *bla*_CTX-M_, *bla*_TEM_,  *bla*_SHV_ | ST117, ST4085, ST1080, ST6496, ST744, ST761, ST5519, ST10*, ST392, ST58*, ST4085, ST410*, ST1121, ST361, ST11, ST69*, ST88* | NT |
| Nakano *et al* [17] | Japan | Cattle (202), swine (93) | COL (25), CTX (50) | PCR | *mcr-1,* *bla*_CTX-M-14_, *bla*_CTX-M-27_, *bla*_CTX-M-156_ | ST2929, ST69*, ST95*, ST106, ST617*, ST10*, ST744, ST746, ST165, ST34, ST398, ST48, ST349, ST5229, ST93, ST88*, ST410*, 1 new ST | *IncF, IncX4, IncX1, IncFIB ,IncFIC, IncI1Iγ, IncHI1, IncY, IncHI2, IncP, IncA/C, IncFIA* |
| Healthy Pets | | | | | | | |
| Habib *et al* [63] | United Arab Emirates | Cats and dogs (77) | COL (2), CTX (70), CRO (73) | WGS | *mcr-1.1,* *bla*_CTX-M-15_, *bla*_TEM-1B_, *bla*_CTX-M-55_, *bla*_TEM-1C_, *bla*_TEM-35_, *bla*_LAP-2_, *bla*_CTX-M14b_, *bla*_CTX-M-2_, *bla*_CMY-2_, *bla*_CTX-M-8_ | ST1011 | *mcr-1* carried by *Incl2* |
| Yousfi *et al* [64] | Algeria | Cats, dogs (173) | CARB (4), CAZ (2), CTX (1) | PCR | *bla*_OXA-48_, *bla*_NDM-5_, *bla*_TEM-1_, *bla*_CTX-M-15_, *bla*_CMY-42_ | NT | NT |

**Abbreviations:** PCR: polymerase chain reaction; WGS: whole-genome sequencing; NT: not tested; NA: not applicable; ST: sequence type; COL: colistin; CARB: carbapenem; FEP: cefepime; CTX: cefotaxime; CAZ: ceftazidime; CRO: ceftriaxone

* = high-risk clones as described by Kocsis *et al* [65].

**References**

1. Li, Y., Ma, L., Ding, X., & Zhang, R. (2024). Fecal carriage and genetic characteristics of carbapenem-resistant enterobacterales among adults from four provinces of China. *Frontiers in epidemiology*, *3*, 1304324. <https://doi.org/10.3389/fepid.2023.1304324>
2. Boonyasiri, A., Brinkac, L. M., Jauneikaite, E., White, R. C., Greco, C., Seenama, C., Tangkoskul, T., Nguyen, K., Fouts, D. E., & Thamlikitkul, V. (2023). Characteristics and genomic epidemiology of colistin-resistant Enterobacterales from farmers, swine, and hospitalized patients in Thailand, 2014-2017. *BMC infectious diseases*, *23*(1), 556. <https://doi.org/10.1186/s12879-023-08539-8>
3. Salimizand, H., Ardalan, F. A., Amini, S., Aminrasouli, H., & Badmasti, F. (2023). Plasmid-borne mobile colistin resistance (MCR-1) in healthy humans and poultry. *International Journal of New Findings in Health and Educational Sciences*, *1*(1), 1–6. <https://doi.org/10.63053/ijhes.3>
4. Yen, N. T. P., Nhung, N. T., Phu, D. H., Dung, N. T. T., Van, N. T. B., Kiet, B. T., Hien, V. B., Larsson, M., Olson, L., Campbell, J., Quynh, N. P. N., Duy, P. T., & Carrique-Mas, J. (2022). Prevalence of carbapenem resistance and its potential association with antimicrobial use in humans and animals in rural communities in Vietnam. *JAC-antimicrobial resistance*, *4*(2), dlac038. <https://doi.org/10.1093/jacamr/dlac038>
5. Bastidas-Caldes, C., Guerrero-Freire, S., Ortuño-Gutiérrez, N., Sunyoto, T., Gomes-Dias, C. A., Ramírez, M. S., Calero-Cáceres, W., Harries, A. D., Rey, J., de Waard, J. H., & Calvopiña, M. (2023). Colistin resistance in *Escherichia coli* and *Klebsiella pneumoniae* in humans and backyard animals in Ecuador. *Revista panamericana de salud publica = Pan American journal of public health*, *47*, e48. <https://doi.org/10.26633/RPSP.2023.48>
6. von Wintersdorff, C. J., Wolffs, P. F., van Niekerk, J. M., Beuken, E., van Alphen, L. B., Stobberingh, E. E., Oude Lashof, A. M., Hoebe, C. J., Savelkoul, P. H., & Penders, J. (2016). Detection of the plasmid-mediated colistin-resistance gene mcr-1 in faecal metagenomes of Dutch travellers. *The Journal of antimicrobial chemotherapy*, *71*(12), 3416–3419. <https://doi.org/10.1093/jac/dkw328>
7. Yamaguchi, T., Kawahara, R., Hamamoto, K., Hirai, I., Khong, D. T., Nguyen, T. N., Tran, H. T., Motooka, D., Nakamura, S., & Yamamoto, Y. (2020). High Prevalence of Colistin-Resistant *Escherichia coli* with Chromosomally Carried *mcr-1* in Healthy Residents in Vietnam. *mSphere*, *5*(2), e00117-20. <https://doi.org/10.1128/mSphere.00117-20>
8. Atterby, C., Osbjer, K., Tepper, V., Rajala, E., Hernandez, J., Seng, S., Holl, D., Bonnedahl, J., Börjesson, S., Magnusson, U., & Järhult, J. D. (2019). Carriage of carbapenemase- and extended-spectrum cephalosporinase-producing *Escherichia coli* and Klebsiella pneumoniae in humans and livestock in rural Cambodia; gender and age differences and detection of bla_OXA-48_ in humans. *Zoonoses and public health*, *66*(6), 603–617. <https://doi.org/10.1111/zph.12612>
9. Yamamoto, Y., Kawahara, R., Fujiya, Y., Sasaki, T., Hirai, I., Khong, D. T., Nguyen, T. N., & Nguyen, B. X. (2019). Wide dissemination of colistin-resistant *Escherichia coli* with the mobile resistance gene mcr in healthy residents in Vietnam. *The Journal of antimicrobial chemotherapy*, *74*(2), 523–524. <https://doi.org/10.1093/jac/dky435>
10. Milenkov, M., Rasoanandrasana, S., Rahajamanana, L. V., Rakotomalala, R. S., Razafindrakoto, C. A., Rafalimanana, C., Ravelomandranto, E., Ravaoarisaina, Z., Westeel, E., Petitjean, M., Mullaert, J., Clermont, O., Raskine, L., Samison, L. H., Endtz, H., Andremont, A., Denamur, E., Komurian-Pradel, F., & Armand-Lefevre, L. (2021). Prevalence, Risk Factors, and Genetic Characterization of Extended-Spectrum Beta-Lactamase *Escherichia coli* Isolated From Healthy Pregnant Women in Madagascar. *Frontiers in microbiology*, *12*, 786146. <https://doi.org/10.3389/fmicb.2021.786146>
11. Zurfluh, K., Nüesch-Inderbinen, M. T., Poirel, L., Nordmann, P., Hächler, H., & Stephan, R. (2015). Emergence of *Escherichia coli* producing OXA-48 β-lactamase in the community in Switzerland. *Antimicrobial resistance and infection control*, *4*, 9. <https://doi.org/10.1186/s13756-015-0051-x>
12. Giani, T., Sennati, S., Antonelli, A., Di Pilato, V., di Maggio, T., Mantella, A., Niccolai, C., Spinicci, M., Monasterio, J., Castellanos, P., Martinez, M., Contreras, F., Balderrama Villaroel, D., Damiani, E., Maury, S., Rocabado, R., Pallecchi, L., Bartoloni, A., & Rossolini, G. M. (2018). High prevalence of carriage of *mcr-1*-positive enteric bacteria among healthy children from rural communities in the Chaco region, Bolivia, September to October 2016. *Euro surveillance : bulletin Europeen sur les maladies transmissibles = European communicable disease bulletin*, *23*(45), 1800115. <https://doi.org/10.2807/1560-7917.ES.2018.23.45.1800115>
13. Chen, B., Berglund, B., Wang, S., Börjesson, S., Bi, Z., Nilsson, M., Yin, H., Zheng, B., Xiao, Y., Bi, Z., & Nilsson, L. E. (2022). Rapid increase in occurrence of carbapenem-resistant Enterobacteriaceae in healthy rural residents in Shandong Province, China, from 2015 to 2017. *Journal of global antimicrobial resistance*, *28*, 38–42. <https://doi.org/10.1016/j.jgar.2021.11.007>
14. Shen, Z., Hu, Y., Sun, Q., Hu, F., Zhou, H., Shu, L., Ma, T., Shen, Y., Wang, Y., Li, J., Walsh, T. R., Zhang, R., & Wang, S. (2018). Emerging Carriage of NDM-5 and MCR-1 in *Escherichia coli* From Healthy People in Multiple Regions in China: A Cross Sectional Observational Study. *EClinicalMedicine*, *6*, 11–20. <https://doi.org/10.1016/j.eclinm.2018.11.003>
15. Mahmoodi, F., Rezatofighi, S. E., & Akhoond, M. R. (2020). Antimicrobial resistance and metallo-beta-lactamase producing among commensal *Escherichia coli* isolates from healthy children of Khuzestan and Fars provinces; Iran. *BMC microbiology*, *20*(1), 366. <https://doi.org/10.1186/s12866-020-02051-8>
16. Al-Mir, H., Osman, M., Drapeau, A., Hamze, M., Madec, J. Y., & Haenni, M. (2021). Spread of ESC-, carbapenem- and colistin-resistant *Escherichia coli* clones and plasmids within and between food workers in Lebanon. *The Journal of antimicrobial chemotherapy*, *76*(12), 3135–3143. <https://doi.org/10.1093/jac/dkab327>
17. Nakano, A., Nakano, R., Nishisouzu, R., Suzuki, Y., Horiuchi, S., Kikuchi-Ueda, T., Ubagai, T., Ono, Y., & Yano, H. (2021). Prevalence and Relatedness of *mcr-1*-Mediated Colistin-Resistant *Escherichia coli* Isolated From Livestock and Farmers in Japan. *Frontiers in microbiology*, *12*, 664931. https://doi.org/10.3389/fmicb.2021.664931
18. Ramatla, T., Tutubala, M., Motlhaping, T., de Wet, L., Mokgokong, P., Thekisoe, O., & Lekota, K. (2024). Molecular detection of Shiga toxin and extended-spectrum beta-lactamase (ESBL)-producing *Escherichia coli* isolates from sheep and goats. *Molecular biology reports*, *51*(1), 57. <https://doi.org/10.1007/s11033-023-08987-0>
19. Ramatla, T., Mokgokong, P., Lekota, K., & Thekisoe, O. (2024). Antimicrobial resistance profiles of *Pseudomonas aeruginosa,* *Escherichia coli* and *Klebsiella pneumoniae* strains isolated from broiler chickens. Food microbiology, 120, 104476. <https://doi.org/10.1016/j.fm.2024.104476>
20. Boonyasiri, A., Brinkac, L. M., Jauneikaite, E., White, R. C., Greco, C., Seenama, C., Tangkoskul, T., Nguyen, K., Fouts, D. E., & Thamlikitkul, V. (2023). Characteristics and genomic epidemiology of colistin-resistant Enterobacterales from farmers, swine, and hospitalized patients in Thailand, 2014-2017. *BMC infectious diseases*, *23*(1), 556. <https://doi.org/10.1186/s12879-023-08539-8>
21. Salimizand, H., Ardalan, F. A., Amini, S., Aminrasouli, H., & Badmasti, F. (2023). Plasmid-borne mobile colistin resistance (MCR-1) in healthy humans and poultry. *International Journal of New Findings in Health and Educational Sciences*, *1*(1), 1–6. https://doi.org/10.63053/ijhes.3
22. Treilles, M., Châtre, P., Drapeau, A., Madec, J. Y., & Haenni, M. (2023). Spread of the *mcr-1* colistin-resistance gene in *Escherichia coli* through plasmid transmission and chromosomal transposition in French goats. *Frontiers in microbiology*, *13*, 1023403. <https://doi.org/10.3389/fmicb.2022.1023403>
23. Yen, N. T. P., Nhung, N. T., Phu, D. H., Dung, N. T. T., Van, N. T. B., Kiet, B. T., Hien, V. B., Larsson, M., Olson, L., Campbell, J., Quynh, N. P. N., Duy, P. T., & Carrique-Mas, J. (2022). Prevalence of carbapenem resistance and its potential association with antimicrobial use in humans and animals in rural communities in Vietnam. *JAC-antimicrobial resistance*, *4*(2), dlac038. <https://doi.org/10.1093/jacamr/dlac038>
24. Bastidas-Caldes, C., Guerrero-Freire, S., Ortuño-Gutiérrez, N., Sunyoto, T., Gomes-Dias, C. A., Ramírez, M. S., Calero-Cáceres, W., Harries, A. D., Rey, J., de Waard, J. H., & Calvopiña, M. (2023). Colistin resistance in *Escherichia coli* and *Klebsiella pneumoniae* in humans and backyard animals in Ecuador. *Revista panamericana de salud publica = Pan American journal of public health*, *47*, e48. <https://doi.org/10.26633/RPSP.2023.48>
25. Peng, Z., Li, X., Hu, Z., Li, Z., Lv, Y., Lei, M., Wu, B., Chen, H., & Wang, X. (2019). Characteristics of Carbapenem-Resistant and Colistin-Resistant *Escherichia coli* Co-Producing NDM-1 and MCR-1 from Pig Farms in China. *Microorganisms*, *7*(11), 482. <https://doi.org/10.3390/microorganisms7110482>
26. Irrgang, A., Roschanski, N., Tenhagen, B. A., Grobbel, M., Skladnikiewicz-Ziemer, T., Thomas, K., Roesler, U., & Käsbohrer, A. (2016). Prevalence of mcr-1 in *E. coli* from Livestock and Food in Germany, 2010-2015. *PloS one*, *11*(7), e0159863. <https://doi.org/10.1371/journal.pone.0159863>
27. Delgado-Blas, J. F., Ovejero, C. M., Abadia-Patiño, L., & Gonzalez-Zorn, B. (2016). Coexistence of mcr-1 and blaNDM-1 in *Escherichia coli* from Venezuela. *Antimicrobial agents and chemotherapy*, *60*(10), 6356–6358. <https://doi.org/10.1128/AAC.01319-16>
28. Rebelo, A. R., Bortolaia, V., Kjeldgaard, J. S., Pedersen, S. K., Leekitcharoenphon, P., Hansen, I. M., Guerra, B., Malorny, B., Borowiak, M., Hammerl, J. A., Battisti, A., Franco, A., Alba, P., Perrin-Guyomard, A., Granier, S. A., De Frutos Escobar, C., Malhotra-Kumar, S., Villa, L., Carattoli, A., & Hendriksen, R. S. (2018). Multiplex PCR for detection of plasmid-mediated colistin resistance determinants, *mcr-1, mcr-2, mcr-3, mcr-4* and *mcr-5* for surveillance purposes. *Euro surveillance : bulletin Europeen sur les maladies transmissibles = European communicable disease bulletin*, *23*(6), 17-00672. <https://doi.org/10.2807/1560-7917.ES.2018.23.6.17-00672>
29. Quesada, A., Ugarte-Ruiz, M., Iglesias, M. R., Porrero, M. C., Martínez, R., Florez-Cuadrado, D., Campos, M. J., García, M., Píriz, S., Sáez, J. L., & Domínguez, L. (2016). Detection of plasmid mediated colistin resistance (MCR-1) in *Escherichia coli* and Salmonella enterica isolated from poultry and swine in Spain. *Research in veterinary science*, *105*, 134–135. <https://doi.org/10.1016/j.rvsc.2016.02.003>
30. Joshi, P. R., Thummeepak, R., Paudel, S., Acharya, M., Pradhan, S., Banjara, M. R., Leungtongkam, U., & Sitthisak, S. (2019). Molecular Characterization of Colistin-Resistant *Escherichia coli* Isolated from Chickens: First Report from Nepal. *Microbial drug resistance (Larchmont, N.Y.)*, *25*(6), 846–854. <https://doi.org/10.1089/mdr.2018.0326>
31. Pulss, S., Semmler, T., Prenger-Berninghoff, E., Bauerfeind, R., & Ewers, C. (2017). First report of an *Escherichia coli* strain from swine carrying an OXA-181 carbapenemase and the colistin resistance determinant MCR-1. *International journal of antimicrobial agents*, *50*(2), 232–236. <https://doi.org/10.1016/j.ijantimicag.2017.03.014>
32. Lengliz, S., Benlabidi, S., Raddaoui, A., Cheriet, S., Ben Chehida, N., Najar, T., & Abbassi, M. S. (2021). High occurrence of carbapenem-resistant *Escherichia coli* isolates from healthy rabbits (Oryctolagus cuniculus): first report of bla_IMI_ and bla_VIM_ type genes from livestock in Tunisia. *Letters in applied microbiology*, *73*(6), 708–717. <https://doi.org/10.1111/lam.13558>
33. Ghazali, M., Chai, M., Sukiman, M., Mohamad, N., & Ariffin, S. Z. (2020). Prevalence of carbapenem-resistant *Escherichia coli* (CREC) within farm animals in Malaysia. *International Journal of Infectious Diseases*, *101*, 534–535. <https://doi.org/10.1016/j.ijid.2020.09.1386>
34. Belaynehe, K. M., Shin, S. W., Park, K. Y., Jang, J. Y., Won, H. G., Yoon, I. J., & Yoo, H. S. (2018). Emergence of mcr-1 and mcr-3 variants coding for plasmid-mediated colistin resistance in *Escherichia coli* isolates from food- producing animals in South Korea. *International journal of infectious diseases : IJID : official publication of the International Society for Infectious Diseases*, *72*, 22–24. <https://doi.org/10.1016/j.ijid.2018.05.011>
35. Hernández, M., Iglesias, M. R., Rodríguez-Lázaro, D., Gallardo, A., Quijada, N., Miguela-Villoldo, P., Campos, M. J., Píriz, S., López-Orozco, G., de Frutos, C., Sáez, J. L., Ugarte-Ruiz, M., Domínguez, L., & Quesada, A. (2017). Co-occurrence of colistin-resistance genes mcr-1 and mcr-3 among multidrug-resistant *Escherichia coli* isolated from cattle, Spain, September 2015. *Euro surveillance : bulletin Europeen sur les maladies transmissibles = European communicable disease bulletin*, *22*(31), 30586. <https://doi.org/10.2807/1560-7917.ES.2017.22.31.30586>
36. Kawanishi, M., Abo, H., Ozawa, M., Uchiyama, M., Shirakawa, T., Suzuki, S., Shima, A., Yamashita, A., Sekizuka, T., Kato, K., Kuroda, M., Koike, R., & Kijima, M. (2016). Prevalence of Colistin Resistance Gene mcr-1 and Absence of mcr-2 in *Escherichia coli* Isolated from Healthy Food-Producing Animals in Japan. *Antimicrobial agents and chemotherapy*, *61*(1), e02057-16. <https://doi.org/10.1128/AAC.02057-16>
37. Alba, P., Leekitcharoenphon, P., Franco, A., Feltrin, F., Ianzano, A., Caprioli, A., Stravino, F., Hendriksen, R. S., Bortolaia, V., & Battisti, A. (2018). Molecular Epidemiology of *mcr*-Encoded Colistin Resistance in *Enterobacteriaceae* From Food-Producing Animals in Italy Revealed Through the EU Harmonized Antimicrobial Resistance Monitoring. *Frontiers in microbiology*, *9*, 1217. <https://doi.org/10.3389/fmicb.2018.01217>
38. Wu, C., Wang, Y., Shi, X., Wang, S., Ren, H., Shen, Z., Wang, Y., Lin, J., & Wang, S. (2018). Rapid rise of the ESBL and mcr-1 genes in *Escherichia coli* of chicken origin in China, 2008-2014. *Emerging microbes & infections*, *7*(1), 30. <https://doi.org/10.1038/s41426-018-0033-1>
39. Maamar, E., Alonso, C. A., Hamzaoui, Z., Dakhli, N., Abbassi, M. S., Ferjani, S., Saidani, M., Boutiba-Ben Boubaker, I., & Torres, C. (2018). Emergence of plasmid-mediated colistin-resistance in CMY-2-producing *Escherichia coli* of lineage ST2197 in a Tunisian poultry farm. *International journal of food microbiology*, *269*, 60–63. <https://doi.org/10.1016/j.ijfoodmicro.2018.01.017>
40. Moawad, A. A., Hotzel, H., Neubauer, H., Ehricht, R., Monecke, S., Tomaso, H., Hafez, H. M., Roesler, U., & El-Adawy, H. (2018). Antimicrobial resistance in *Enterobacteriaceae* from healthy broilers in Egypt: emergence of colistin-resistant and extended-spectrum β-lactamase-producing *Escherichia coli*. *Gut pathogens*, *10*, 39. <https://doi.org/10.1186/s13099-018-0266-5>
41. Hassen, B., Abbassi, M. S., Ruiz-Ripa, L., Mama, O. M., Hassen, A., Torres, C., & Hammami, S. (2020). High prevalence of mcr-1 encoding colistin resistance and first identification of bla_CTX-M-55_ in ESBL/CMY-2-producing *Escherichia coli* isolated from chicken faeces and retail meat in Tunisia. *International journal of food microbiology*, *318*, 108478. <https://doi.org/10.1016/j.ijfoodmicro.2019.108478>
42. Büdel, T., Kuenzli, E., Campos-Madueno, E. I., Mohammed, A. H., Hassan, N. K., Zinsstag, J., Hatz, C., & Endimiani, A. (2020). On the island of Zanzibar people in the community are frequently colonized with the same MDR Enterobacterales found in poultry and retailed chicken meat. *The Journal of antimicrobial chemotherapy*, *75*(9), 2432–2441. <https://doi.org/10.1093/jac/dkaa198>
43. Dominguez, J. E., Redondo, L. M., Figueroa Espinosa, R. A., Cejas, D., Gutkind, G. O., Chacana, P. A., Di Conza, J. A., & Fernández Miyakawa, M. E. (2018). Simultaneous Carriage of *mcr-1* and Other Antimicrobial Resistance Determinants in *Escherichia coli* From Poultry. *Frontiers in microbiology*, *9*, 1679. <https://doi.org/10.3389/fmicb.2018.01679>
44. Vounba, P., Rhouma, M., Arsenault, J., Bada Alambédji, R., Fravalo, P., & Fairbrother, J. M. (2019). Prevalence of colistin resistance and mcr-1/mcr-2 genes in extended-spectrum β-lactamase/AmpC-producing *Escherichia coli* isolated from chickens in Canada, Senegal and Vietnam. *Journal of global antimicrobial resistance*, *19*, 222–227. <https://doi.org/10.1016/j.jgar.2019.05.002>
45. Yamamoto, Y., Calvopina, M., Izurieta, R., Villacres, I., Kawahara, R., Sasaki, M., & Yamamoto, M. (2019). Colistin-resistant *Escherichia coli* with mcr genes in the livestock of rural small-scale farms in Ecuador. *BMC research notes*, *12*(1), 121. <https://doi.org/10.1186/s13104-019-4144-0>
46. Nesporova, K., Jamborova, I., Valcek, A., Medvecky, M., Literak, I., & Dolejska, M. (2019). Various conjugative plasmids carrying the mcr-5 gene in *Escherichia coli* isolates from healthy chickens in Paraguay. *The Journal of antimicrobial chemotherapy*, *74*(11), 3394–3397. <https://doi.org/10.1093/jac/dkz317>
47. G., Casaux, M. L., Fraga, M., Zunino, P., Bado, I., & Vignoli, R. (2020). Transferable Resistance to Highest Priority Critically Important Antibiotics for Human Health in *Escherichia coli* Strains Obtained From Livestock Feces in Uruguay. *Frontiers in veterinary science*, *7*, 588919. <https://doi.org/10.3389/fvets.2020.588919>
48. Eltai, N. O., Abdfarag, E. A., Al-Romaihi, H., Wehedy, E., Mahmoud, M. H., Alawad, O. K., Al-Hajri, M. M., Al Thani, A. A., & Yassine, H. M. (2018). Antibiotic Resistance Profile of Commensal *Escherichia coli* Isolated from Broiler Chickens in Qatar. *Journal of food protection*, *81*(2), 302–307. <https://doi.org/10.4315/0362-028X.JFP-17-191>
49. Hmede, Z., & Kassem, I. I. (2018). The Colistin Resistance Gene *mcr-1* Is Prevalent in Commensal *Escherichia coli* Isolated from Preharvest Poultry in Lebanon. *Antimicrobial agents and chemotherapy*, *62*(11), e01304-18. <https://doi.org/10.1128/AAC.01304-18>
50. Veldman, K., van Essen-Zandbergen, A., Rapallini, M., Wit, B., Heymans, R., van Pelt, W., & Mevius, D. (2016). Location of colistin resistance gene mcr-1 in Enterobacteriaceae from livestock and meat. *The Journal of antimicrobial chemotherapy*, *71*(8), 2340–2342. <https://doi.org/10.1093/jac/dkw181>
51. Lv, J., Mohsin, M., Lei, S., Srinivas, S., Wiqar, R. T., Lin, J., & Feng, Y. (2018). Discovery of a mcr-1-bearing plasmid in commensal colistin-resistant *Escherichia coli* from healthy broilers in Faisalabad, Pakistan. *Virulence*, *9*(1), 994–999. <https://doi.org/10.1080/21505594.2018.1462060>
52. Zhang, X., Zhang, B., Guo, Y., Wang, J., Zhao, P., Liu, J., & He, K. (2019). Colistin resistance prevalence in *Escherichia coli* from domestic animals in intensive breeding farms of Jiangsu Province. *International journal of food microbiology*, *291*, 87–90. <https://doi.org/10.1016/j.ijfoodmicro.2018.11.013>
53. Zhang, J., Chen, L., Wang, J., Yassin, A. K., Butaye, P., Kelly, P., Gong, J., Guo, W., Li, J., Li, M., Yang, F., Feng, Z., Jiang, P., Song, C., Wang, Y., You, J., Yang, Y., Price, S., Qi, K., Kang, Y., … Wang, C. (2018). Molecular detection of colistin resistance genes (mcr-1, mcr-2 and mcr-3) in nasal/oropharyngeal and anal/cloacal swabs from pigs and poultry. *Scientific reports*, *8*(1), 3705. <https://doi.org/10.1038/s41598-018-22084-4>
54. Oh, S. S., Song, J., Kim, J., & Shin, J. (2020). Increasing prevalence of multidrug-resistant mcr-1-positive *Escherichia coli* isolates from fresh vegetables and healthy food animals in South Korea. *International journal of infectious diseases : IJID : official publication of the International Society for Infectious Diseases*, *92*, 53–55. <https://doi.org/10.1016/j.ijid.2019.12.025>
55. Kawahara, R., Fujiya, Y., Yamaguchi, T., Khong, D. T., Nguyen, T. N., Tran, H. T., & Yamamoto, Y. (2019). Most Domestic Livestock Possess Colistin-Resistant Commensal *Escherichia coli* Harboring *mcr* in a Rural Community in Vietnam. *Antimicrobial agents and chemotherapy*, *63*(6), e00594-19. <https://doi.org/10.1128/AAC.00594-19>
56. Jalil, A., Gul, S., Bhatti, M. F., Siddiqui, M. F., & Adnan, F. (2022). High Occurrence of Multidrug-Resistant *Escherichia coli* Strains in Bovine Fecal Samples from Healthy Cows Serves as Rich Reservoir for AMR Transmission. *Antibiotics (Basel, Switzerland)*, *12*(1), 37. <https://doi.org/10.3390/antibiotics12010037>
57. Zou, M., Ma, P. P., Liu, W. S., Liang, X., Li, X. Y., Li, Y. Z., & Liu, B. T. (2021). Prevalence and Antibiotic Resistance Characteristics of Extraintestinal Pathogenic *Escherichia coli* among Healthy Chickens from Farms and Live Poultry Markets in China. *Animals : an open access journal from MDPI*, *11*(4), 1112. <https://doi.org/10.3390/ani11041112>
58. Ahmed, S., Das, T., Islam, M. Z., Herrero-Fresno, A., Biswas, P. K., & Olsen, J. E. (2020). High prevalence of mcr-1-encoded colistin resistance in commensal *Escherichia coli* from broiler chicken in Bangladesh. *Scientific reports*, *10*(1), 18637. <https://doi.org/10.1038/s41598-020-75608-2>
59. Chabou, S., Leulmi, H., & Rolain, J. M. (2019). Emergence of mcr-1-mediated colistin resistance in *Escherichia coli* isolates from poultry in Algeria. *Journal of global antimicrobial resistance*, *16*, 115–116. <https://doi.org/10.1016/j.jgar.2018.12.012>
60. Murugan MS, Sinha DK, Vinodh Kumar OR, Yadav AK, Pruthvishree BS, Vadhana P, Nirupama KR, Bhardwaj M, Singh BR. Epidemiology of carbapenem-resistant Escherichia coli and first report of blaVIM carbapenemases gene in calves from India. Epidemiol Infect. 2019 Jan;147:e159. doi: 10.1017/S0950268819000463. PMID: 31063112; PMCID: PMC6518490.
61. De Koster S, Ringenier M, Xavier BB, Lammens C, De Coninck D, De Bruyne K, Mensaert K, Kluytmans-van den Bergh M, Kluytmans J, Dewulf J, Goossens H; i-4-1-Health Study Group. Genetic characterization of ESBL-producing and ciprofloxacin-resistant *Escherichia coli* from Belgian broilers and pigs. Front Microbiol. 2023 Apr 6;14:1150470. doi: 10.3389/fmicb.2023.1150470. PMID: 37089550; PMCID: PMC10116946.
62. Shafiq, M., Rahman, S. U., Bilal, H., Ullah, A., Noman, S. M., Zeng, M., Yuan, Y., Xie, Q., Li, X., & Jiao, X. (2022). Incidence and molecular characterization of ESBL-producing and colistin-resistant *Escherichia coli* isolates recovered from healthy food-producing animals in Pakistan. *Journal of applied microbiology*, *133*(3), 1169–1182. <https://doi.org/10.1111/jam.15469>
63. Habib, I., Elbediwi, M., Mohteshamuddin, K., Mohamed, M. I., Lakshmi, G. B., Abdalla, A., Anes, F., Ghazawi, A., Khan, M., & Khalifa, H. (2023). Genomic profiling of extended-spectrum β-lactamase-producing *Escherichia coli* from Pets in the United Arab Emirates: Unveiling colistin resistance mediated by mcr-1.1 and its probable transmission from chicken meat - A One Health perspective. *Journal of infection and public health*, *16 Suppl 1*, 163–171. <https://doi.org/10.1016/j.jiph.2023.10.034>
64. Yousfi, M., Touati, A., Mairi, A., Brasme, L., Gharout-Sait, A., Guillard, T., & De Champs, C. (2016). Emergence of Carbapenemase-Producing *Escherichia coli* Isolated from Companion Animals in Algeria. *Microbial drug resistance (Larchmont, N.Y.)*, *22*(4), 342–346. <https://doi.org/10.1089/mdr.2015.0196>
65. Kocsis, B., Gulyás, D., Szabó, D. Emergence and Dissemination of Extraintestinal Pathogenic High-Risk International Clones of *Escherichia coli*. Life 2022, 12, 2077. https://doi.org/10.3390/life12122077
